# The Biology and Function of Extracellular Vesicles in Cancer Development

**DOI:** 10.3389/fcell.2021.777441

**Published:** 2021-11-05

**Authors:** Xinyi Zhang, Dianfeng Liu, Yongjian Gao, Chao Lin, Qingwu An, Ye Feng, Yangyang Liu, Da Liu, Haoming Luo, Dongxu Wang

**Affiliations:** ^1^ Laboratory Animal Center, College of Animal Science, Jilin University, Changchun, China; ^2^ Department of Hepatobiliary and Pancreas Surgery, China–Japan Union Hospital of Jilin University, Changchun, China; ^3^ Department of Pharmacy, Changchun University of Chinese Medicine, Changchun, China; ^4^ School of Grain Science and Technology, Jilin Business and Technology College, Changchun, China

**Keywords:** EVS, Cancer, ncRNA, drug loading, target

## Abstract

Extracellular vesicles (EVs) exert their biological functions by delivering proteins, metabolites, and nucleic acids to recipient cells. EVs play important roles in cancer development. The anti-tumor effect of EVs is by their cargos carrying proteins, metabolites, and nucleic acids to affect cell-to-cell communication. The characteristics of cell-to-cell communication can potentially be applied for the therapy of cancers, such as gastric cancer. In addition, EVs can be used as an effective cargos to deliver ncRNAs, peptides, and drugs, to target tumor tissues. In addition, EVs have the ability to regulate cell apoptosis, autophagy, proliferation, and migration of cancer cells. The ncRNA and peptides that were engaged with EVs were associated with cell signaling pathways in cancer development. This review focuses on the composition, cargo, function, mechanism, and application of EVs in cancers.

## Introduction

EVs are 40–100 nm extracellular vesicles that are released by cells (Kahlert and Kalluri, 2013). EVs were initially observed in sheep reticulocytes in the 1980s (Raposo and Stoorvogel, 2013). Recently, studies have focused on the source of their endocytosis and on distinguishing them from micro-vesicles (Théry et al., 2002). EVs have anti-tumor functions associated with the development of a variety of cancers, such as breast, stomach, liver, and lung cancers (Table 1).

**TABLE 1:** The function of EVs in cancers.

Name	Fatality rate (%)	Function of EVs	References
Lung cancer	89	Diagnosis	Kahlert and Kalluri, (2013)
Liver cancer	60–70	Inhibited cell growth	Raposo and Stoorvogel, (2013)
gastric cancer	12.4	Induce cell apoptosis	Théry et al. (2002)
Colon cancer	12	Inhibited EMT	Kowal et al. (2016)
Breast cancer	6.6	Plasma biomarkers	Kahlert et al. (2014)

### The Biogenesis and Composition of EVs

Mammalian cell, EVs are highly heterogeneous. They contain lipid membranes, proteins, RNAs, and DNAs (Kowal et al., 2016). The lipid membrane of EVs carries the ligands and receptors from the source cells and has a role in cell-to-cell communication (Valadi et al., 2007; Kahlert et al., 2014). Due to the specificity of the lipid membrane, EVs can invade target cells through biogenesis (Balaj et al., 2011). The components on the membrane also play a key role in cell-to-cell communication (Wu et al., 2021). EVs use lipid membranes to enter recipient cells to release cargo and affect recipient cells. These characteristics indicate that EVs have potential applications in regulating cancer development.

### The Formation of EVs

**FIGURE 1:**
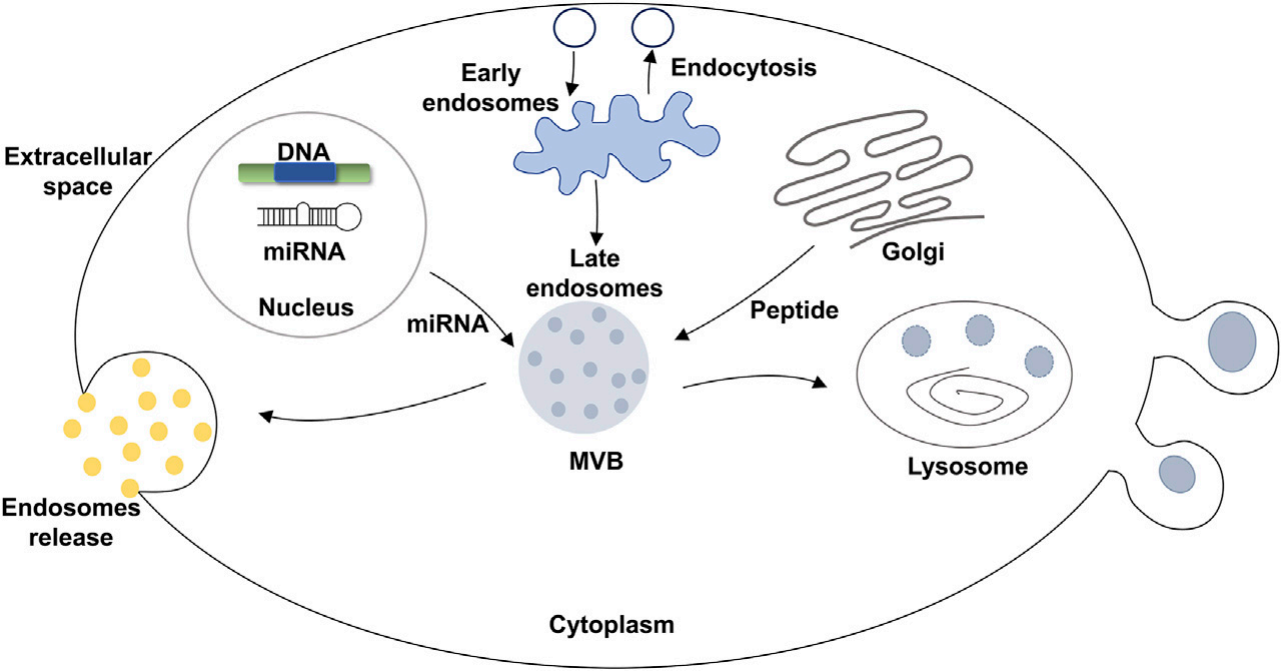
Formation of EVs.

Many EVs formed from normal and pathological cells. In contrast to micro-vesicles, EVs are mainly derived from multivesicular bodies (MVBs) that are formed by intracellular lysosomal particles. EVs are released into the extracellular matrix through the fusion of the outer membrane of the MVBs with the membrane of source cells (Figure 1). Specifically, EVs are formed through the endosomal pathway. First, the endosome is formed by the invasion of the plasma membrane during cell maturation process (Harding et al., 1983). The endosome is a membrane-encapsulated vesicular structure and includes both early and late endosomes. Early endosomes are usually located outside of the cytoplasm. In contrast, late endosomes are located inside of the cytoplasm, near the nucleus. Endosomes are acidic vesicles without lysosomal enzymes (Bainton and Farquhar, 1968). The invasion of endosomes produces MVBs which contain 40–150 nm vesicles. The inner membrane forms intraluminal vesicles (ILV). Finally, the late lysosome melts or fuses with the plasma membrane of the source cell and degrades MVBS to release EVs (Harding et al., 1983). This process is known as EV biogenesis and is different from apoptotic bodies (Taylor and Gercel-Taylor, 2008). EVs are widely observed in tumor cells, mesenchymal stem cells, fibroblasts, neurons, endothelial cells (ECs), and epithelial cells (Kalluri, 2016). Previous reports have suggested that the tumor cells can specifically absorb their own secreted EVs (Kahlert and Kalluri, 2013). This implies that during the formation of EVs, specific biomarkers are formed on the surface of the EVs. These biomarkers are the cues that render EVs to be absorbed by specific cells.

### EVs Cargo

**FIGURE 2:**
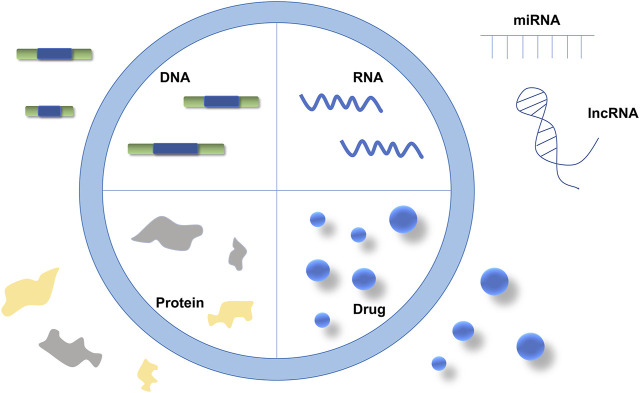
The contents of EVs.

Nucleic acids such as DNAs or RNAs, proteins, or drugs can be carried in EVs as cargo to be delivered for cell-to-cell communication (Figure 2). In the past decades, miRNAs and mRNAs have been found to be major components of EVs. The improvement of EV detection techniques has allowed more RNA species, including transfer RNAs (tRNAs), long non-coding RNAs (lncRNAs), and viral RNAs, to be observed (Valadi et al., 2007; Su et al., 2021). An increasing amount of data suggests that these RNAs, such as lncRNA, have crucial functions that affect the development of cancer cells (Gusachenko et al., 2013). Moreover, numerous studies have demonstrated that the abnormal expressions of miRNAs, lncRNAs, and mRNAs are associated with cancer development (Chan and Tay, 2018; Huang et al., 2020). Hence, these RNAs, that are contained within EVs, can either preserve or degrade their target genes.

Cancers develop because of the expression and interaction of numerous genes or proteins. EVs can express proteins through genetic engineering (Silva et al., 2021). The EVs were obtained from the source cells that were transfected with the target gene plasmids. These EVs contain the synthesized proteins or peptides through cell culture (Perin et al., 2011). There is evidence that fusing the exosomally-enriched membrane protein (Lamp 2b) with the ischemic myocardium‐targeting peptide (IMTP) can be used to inhibit cancer development by molecular cloning lentiviral packaging protocols (Fernández et al., 2002). EVs secreted by tumor cells can be taken up by the same tumor cell with specificity. Some molecules (such as Let-7a) can be easily introduced to donor cells through EVs, and tumor targeting EVs carrying these molecules can be used for cancer treatment (Wu et al., 2021). In addition, EVs can carry various chemotherapeutic drugs and materials for targeted treatment of cancers (Wang et al., 2019a).

### EVs can Decide Cell Fate

**FIGURE 3:**
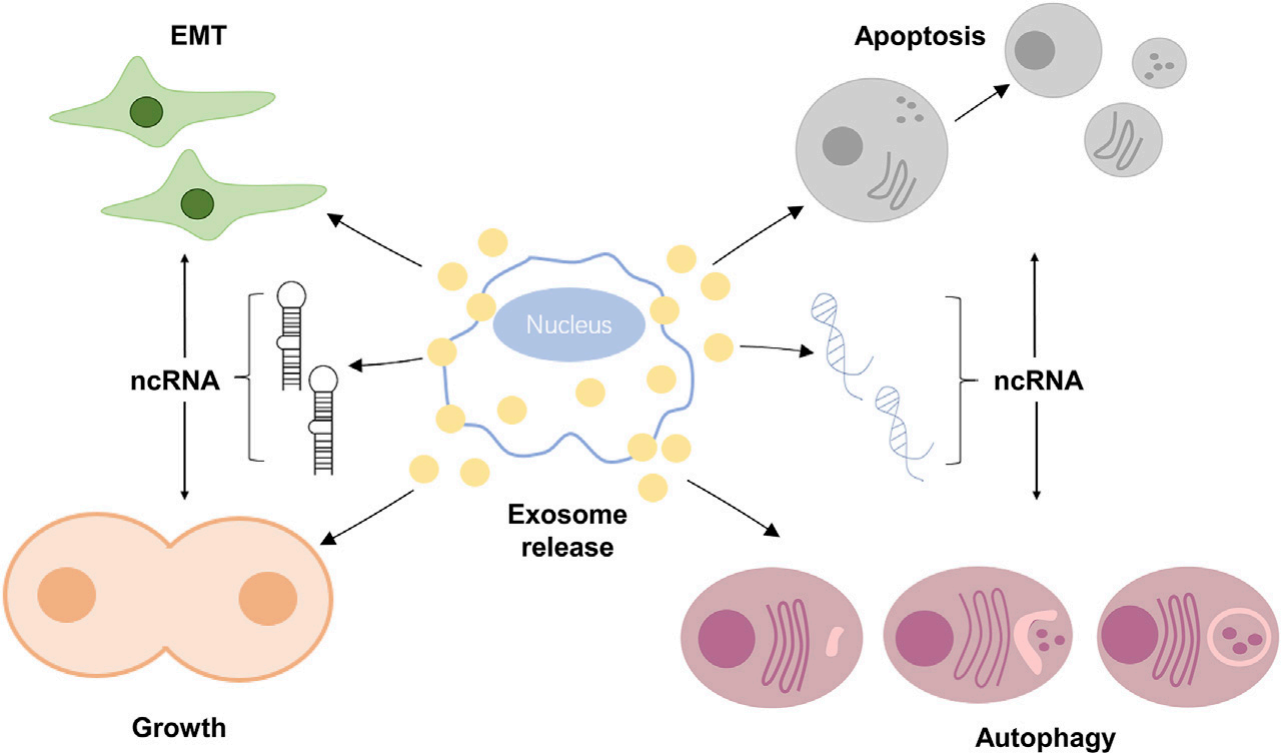
EVs decide cell fate.

The function of EVs depends on the source cells, such as tumor cells or stem cells (Draganov et al., 2019; Dzobo et al., 2020). The EVs released from these source cells can affect the apoptosis, growth, cell cycle, migration, invasion, and differentiation of recipient cells. Previous studies have indicated that tumor-released EVs could deliver genetic information to the recipient cells for cell-to-cell communication (Valadi et al., 2007). This process promotes cell growth, invasion, and active angiogenesis in a tumor microenvironment (Figure 3).

Initially, EVs were considered to be “garbage bags” that could not affect other cells (Kalluri, 2016). However, it was found that EVs could be absorbed by target cells and their cargos could be released to affect cell signaling transduction, therefore determining the fate of the recipient cells (Pan et al., 1985). Additional evidence suggested that tumor cells released EVs that promoted tumor growth and invasion *in vivo* (Ramírez-Ricardo et al., 2020). EVs that carried tumor suppressors, such as let-7a, could inhibited tumor growth (Melo et al., 2014).

### The Function of EVs in Cell Proliferation

Indefinite proliferation is a key feature of tumor cells. The abnormal cell cycle of tumor cells is associated with un-controlled cell growth. Previous reports confirmed that miRNA-122 was involved in the cell cycle as well as the proliferation of hepatocellular carcinoma (HCC) cells (Fernández et al., 2002; Xu et al., 2011). A recent report showed that the EVs carrying circRNA plays a role in the proliferation of HCC cells (Xue et al., 2017). In addition, arsenite could increase the expression of circRNA_100284 carried by EVs, altering the cell cycle and their proliferation by acting on miR-217 (Lu et al., 2015). The expression of the cell proliferation biomarkers E2H2 and cyclin D1 were regulated by the circRNA_100284 contained within EVs, and the expression of circRASSF2 was increased in laryngeal squamous cell carcinoma (LSCC) tissue compared to paracancerous tissue. The circRASSF2 carried by EVs promoted LSCC cell growth via the miR-302B-3p/IGF-1R axis (Tian et al., 2019). Thus, EVs have the ability to regulate cell proliferation through their cargos.

### The Function of EVs in Epithelial-Mesenchymal Transition

The cell-to-cell communication in tumors might promote EMT of cancers. Previous data has shown that the EV-released circRNA PED8A was associated with increased lymphatic invasion, TNM staging, and low survival rate of patients. Furthermore, the circRNA PED8A from EVs promoted tumor cell growth by activating MET, which is a tyrosine kinase receptor (Luna et al., 2019). In addition, the release of circRNA PED8A contained within EVs into the blood circulation promotes invasion and metastasis through the MACC-MET-ERK or AKT pathway. More evidence indicated that EV-released circRNA NRIP1 promoted proliferation, migration, and metastasis through AKT1/mTOR signaling pathway in gastric cancer. The involvement of this pathway has also been confirmed in breast cancer cells in patients (Wang et al., 2019b; Zhang et al., 2019). The circPTGR1 carried in EVs was found to contribute to the metastasis of hepatocellular carcinoma (Wang et al., 2019c). Interestingly, knock out of circPTGR1 in the source cells, their EVs inhibited invasion and migration of cancer cells. The increased expression of EV-released circ-IARS is related to the EMT of pancreatic cancer (Li et al., 2018). Therefore, EVs can act as messenger vehicles for cell-to-cell communication, releasing ncRNAs that contribute to the EMT in cancers.

### The Function of EVs in Apoptosis and Autophagy

Cell apoptosis and autophagy are programmed cell death, both of them are abnormal in cancers. Previous reports have indicated that EVs containing anti-tumor drugs can induce cell apoptosis in HCCs (Slomka et al., 2020). Furthermore, EVs containing miRNA mimics such as let-7a have been found to induce cell apoptosis in breast cancer (Ahmed et al., 2021). In addition, EVs have the ability to regulate autophagy. There is evidence that EVs can enhance autophagy in glioblastoma (GBM) (Pavlyukov et al., 2018). These findings suggest that EVs play a role in cell apoptosis and autophagy.

### EVs Stimulate Oxidative Stress

**FIGURE 4:**
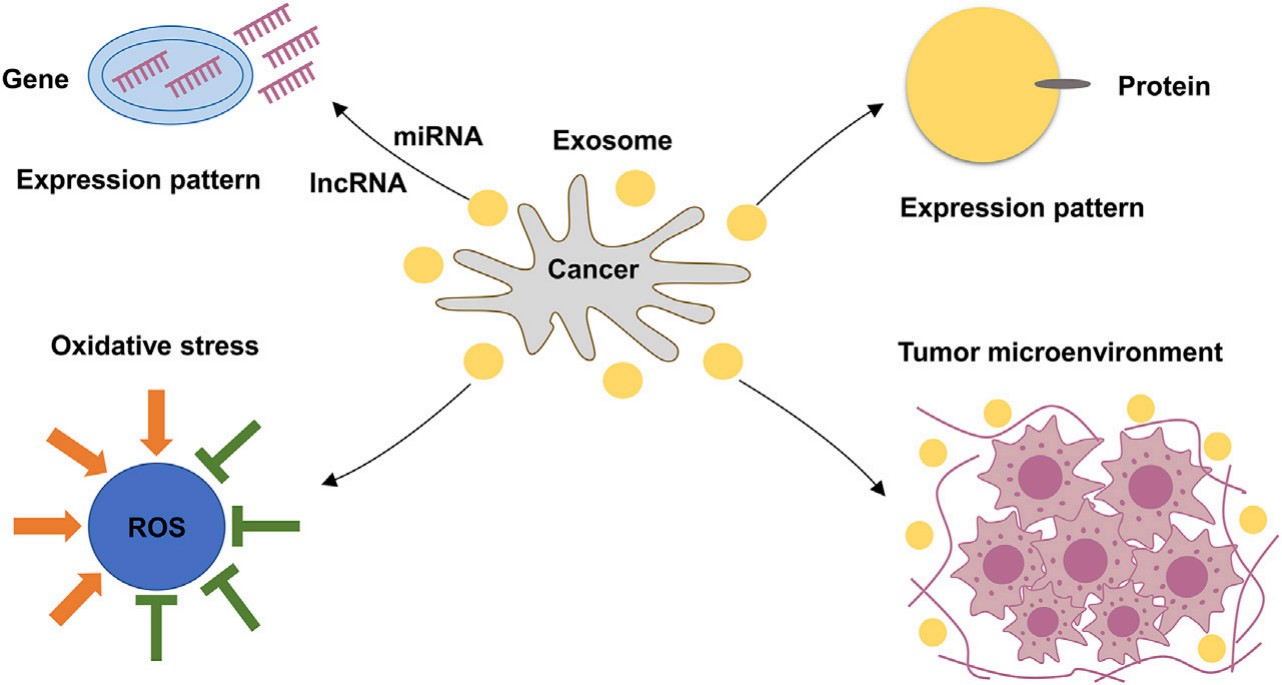
The function of EVs.

Studies have shown that low levels of reactive oxygen species (ROS) were observed in the stem cells of liver cancer and breast cancer (Shi et al., 2012). The EVs of SV-HUC-1 cells were found to mediate the P38/NF-kB signaling pathway, enhancing the levels of OS (Xi et al., 2020). This suggests that EVs were involved in OS, that may contribute to the development of cancers (Figure 4).

### EVs Regulate the Expression of lncRNA

LncRNA usually acts as a regulator of nuclear transcription factors (Wu et al., 2021). An increasing amount of data has shown that long non-coding RNAs (lncRNAs) are associated with the development of cancers (Huang et al., 2021a). EVs containing lncRNA-APC1 inhibited tumor growth in colorectal cancer (CRC). lncRNA-APC1 is an important mediator of APC development through the APC1/RAB5B axis (Wang et al., 2021). The increased expression of lncRNA H19, which is normally regulated by DNA methylation, was observed in numerous cancers (Yang et al., 2021). Previous studies have suggested that EV-contained H19 promotes cell migration and invasion in CRC (Ren et al., 2018). The abnormal expression of *XIST*, a key factor in the X chromosome inactive (XCI) process, was observed in gastric cancer (Chen et al., 2016; Huang et al., 2021a; Huang et al., 2021b). EV-contained *XIST* was found to stimulate cell growth in breast cancer (Xing et al., 2018).

To investigate the role of EVs that contained lncRNAs in cancers, appropriate EVs were collected. The EVs were mostly obtained from the cells that were enriched in expressed lncRNA, such as the A549 cell line which exhibited increased H19 expression (Hao et al., 2017). In addition, the EVs were cultured in an environment that encouraged the increased expression of lncRNAs (Born et al., 2020).

### EVs Regulate the Expression of miRNA

In contrast to lncRNAs, miRNAs are 20–22 nucleotides long. Both miRNAs and lncRNAs are single-stranded, endogenous RNAs, and play roles in the development of cancers. Some miRNAs, such as let-7a and the miR29 family, are involved in EMT, metastasis, migration, invasion, cell cycle, proliferation, and apoptosis of numerous cancers (Rostas et al., 2014; Song et al., 2020). A few miRNAs have been confirmed to be post-transcriptional regulators for target mRNAs. They can be used as the potential biomarkers for classification, prognosis, chemotherapy, and radiotherapy resistance in triple-negative breast cancer (TNBC) (Ding et al., 2019). Results show that miRNA of EVs have a curing effect on breast cancer (Ohno et al., 2013). MiRNAs can be coated by EVs and delivered to target cells, affect the H19/MAPK/ERK pathways (Ding et al., 2018; Wu et al., 2021).

A database indicated that EVs are enriched in miRNAs, lncRNAs, and proteins (Berardocco et al., 2017). In contrast to transfected mimics or miRNAs inhibitors, EVs that obtained from source cells can specifically and accurately deliver these miRNAs endogenously (Table 2). Considering the characteristics of EVs, therapies using EVs could be a potential approach for cancer treatment.

**TABLE 2:** The miRNA of EVs in cancers.

EVs source	miRNA	Mimics/Inhibitor	Function	Cancer	References
LIM1863 cells	miR-106b-3p	Mimics	Inhibits cell growth	CRC	Valadi et al. (2007)
LIM1863 cells	miR-126–3p	Inhibitor	Inhibits metastasis	Breast cancer	Balaj et al. (2011)
LIM1863 cells	miR-126–5p	Mimics	Inhibits EMT	Prostate cancer	Wu et al. (2021)
LIM1863 cells	miR-355–3p	Mimics	Inhibits cell growth	CRC	Harding et al. (1983)
Urine	FOLH1	Mimics	Diagnostic	Prostate cancer	Bainton and Farquhar, (1968)
Urine	HPN	Mimics	Diagnostic	Prostate cancer	Bainton and Farquhar, (1968)
Urine	ITSN1	Mimics	Diagnostic	Prostate cancer	Bainton and Farquhar, (1968)
Urine	CFD miR-21	Inhibitor	Diagnostic	Prostate cancer	Bainton and Farquhar, (1968)
PDAC cell lines	miR-195	Mimics	Diagnostic	PDAC	Taylor and Gercel-Taylor, (2008)
PDAC cell lines		Mimics	Diagnostic	PDAC	Taylor and Gercel-Taylor, (2008)

### EVs Regulate Gene Expression by siRNA

SiRNAs are produced by short, exogenous double-stranded RNAs (dsRNAs) as an RNA interference (RNAi) tool (Kim et al., 2018; Dharamdasani et al., 2020; Feng et al., 2020). SiRNA can be used to effectively silence target genes. A recent study showed that the use of siRNA, such as siRNA-027 can inhibit cell growth and induce apoptosis in numerous cancers (Chen et al., 2020). Hence, siRNA can be used to potentially analyze the development of cancers. A barrier to the RNAi-based therapy of cancers is the low specificity of siRNA delivery. EVs are nano-scale vesicles that can be used to deliver siRNAs as cargos to the target cells by cell-to-cell communication. Previous reports have suggested that the EVs of human plasma cells can deliver siRNA to monocytes and lymphocytes that can silence the expression of mitogen-activated protein kinase 1 (Wahlgren et al., 2012). This suggests that EVs can be used as gene delivery vehicles (GDV) to transport exogenous siRNA in cancer research. Consequently, EVs combined with siRNA are more effective and demonstrate higher specificities than traditionally siRNA delivery in cancer treatment.

### EVs Regulate the Expression of Protein

The mitochondrial proteins contained in EVs can promote tumorigenesis by cell-to-cell communication (Al-Nedawi et al., 2008; Demory Beckler et al., 2013). The expression of MET (also known as hepatocyte growth factor receptors) associated with circulating EVs and phosphorylated MET (Tyr1349) was increased in patients with stage 3 and stage 4 melanoma compare to control (Peinado et al., 2012). This finding indicates that EVs can be used to detect the development of cancer (Costa-Silva et al., 2015). This assumption was confirmed when the expression of MIF and GPC-1 proteins in EVs was detected in cancer patients, allowing them to analyze the prognosis of cancer (Melo et al., 2015). Furthermore, phospholipid-binding proteins-carrying EVs can inhibit cell growth and induced apoptosis in numerous cancers (Dhondt et al., 2020). Thus, the proteins contained in EVs were useful for the detection and prognosis of cancers.

### The Function of EVs in the Tumor Micro-environment

EVs are a key component of the tumor microenvironment. Tumor heterogeneity includes genomic heterogeneity in both tumor cells and non-cancerous microenvironments. Moreover, the tumor nanoenvironment (TNE) is a special nano-scale tumor microenvironment that possesses complex structures and unique components (Eguchi et al., 2018). The TNE includes EVs and apoptotic bodies. EVs released by tumor cells were absorbed by other cells in the tumor microenvironment, influencing the development of cancer through tumor heterogeneity (Tredan et al., 2007). EVs thus contribute to the formation of the tumor microenvironment in the form of cell-to-cell communication.

## Discussion

Considering that EVs can carry any cargos, including nucleic acids and proteins, EVs can thus be used as clinical diagnostic biomarkers. For example, the detection of tumor-specific RNAs in EVs can be used as biomarkers for cancer diagnosis (Gurunathan et al., 2019). Furthermore, proteins contained within EVs such as TSG101, RAS-related protein RAB-11B (RAB11B), CD63, and CD81 can be used as biomarkers for diagnosis of HCCs and other cancers (Möbius et al., 2003; Valadi et al., 2007). In contrast to traditional diagnostic methods such as peripheral blood or histopathology, the accuracy and specificity of EVs were more closely associated with the development of cancers.

EVs can be combined with engineered materials to specifically affect cancer cells. Gold nanoparticles (AuNPs) can mediate photothermal therapy (PPT) to inhibit cell growth and induce cell death (Hu et al., 2020). However, most AuNPs have low specificity. EVs combined with AuNPs can increase their specificity and accelerate the release of their cargos, enhancing the anti-tumor effect of PTT (Nasseri et al., 2020). This could be an important form of therapy for the treatment of cancers in the future. Due to the endogenous nature of EVs, their cargos can escape the immune system and accurately and effectively target tumor cells. In addition, as nano-vesicles, EVs can bypass the blood-brain barrier (Yin et al., 2012). The EVs of immature dendritic cells have been engineered to contain proteins that can target tumors originated from the neuroendothelial and nerve cells in the brain (Federici et al., 2014). Therefore, EVs as nano-vesicles can be used to cross the blood-brain barrier in cancer treatment.

EVs containing anti-cancer drugs, such as therapeutic agents, can be used in the treatment of cancers. In contrast to liposomes, EVs injected *in vivo* can be absorbed without the interference of the immune system (Ferguson and Nguyen, 2016; Kalluri, 2016; Barile and Vassalli, 2017; Fitts et al., 2019; Liao et al., 2019). Furthermore, EVs are safe and are tolerable *in vivo*. Recent studies have demonstrated that repeatedly injected mesenchymal cells (MHC) or the IPCs of EVs do not induce toxicity (Zhu et al., 2017; Mendt et al., 2018).

**FIGURE 5:**
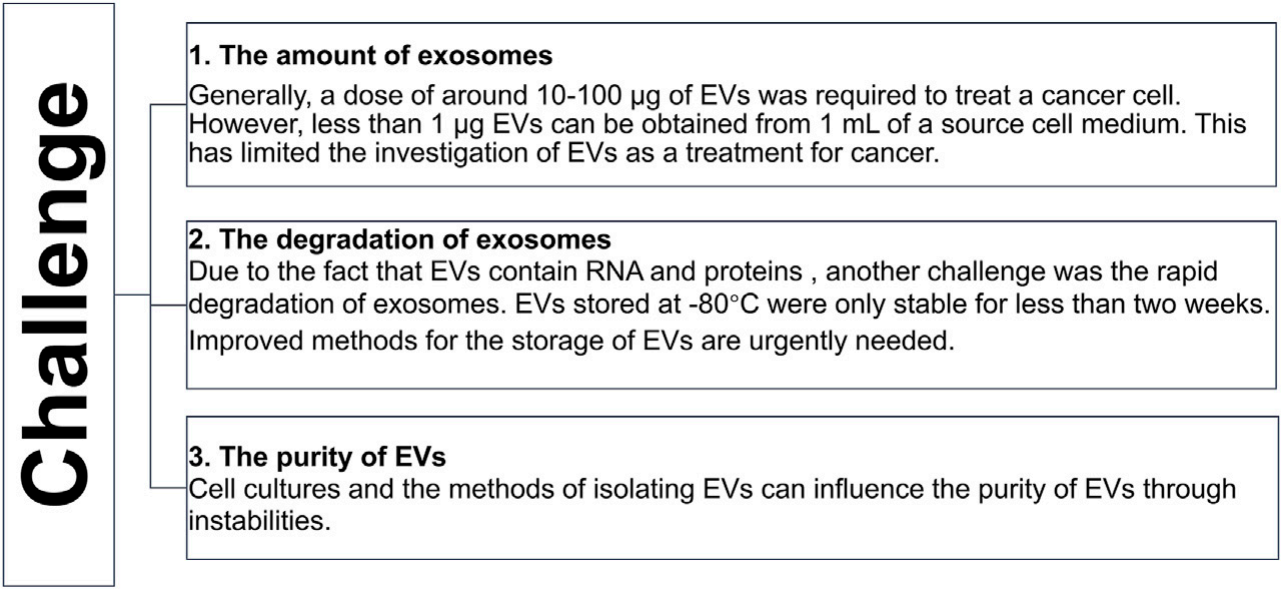
The challenge of EVs.

The EVs that carry chemotherapeutics can decide the cell fate by cell-to-cell communication. For example, αv integrin-specific EVs have been shown to have a therapeutic effect on breast cancer (Tian et al., 2014). Another report suggested that paclitaxel surrounding the EVs of macrophages inhibited lung cancer growth in mice (Kim et al., 2016). These reports indicated that chemotherapeutic agent encapsulating EVs have an anti-tumor effect. Recently, studies have shown that the bioavailability of EVs-engineered doxorubicin was improved compared to the free doxorubicin (Tian et al., 2014; Kojima et al., 2018). These studies suggested that as a vesicle, EVs can enhance the efficacy of drugs. Despite the advancements in the understanding of EVs, there are still some challenges that need to be solved (Figure 5).

## Conclusion

EVs are derived from multivesicular bodies formed by intracellular lysosomal particles that are released into the extracellular matrix. The source cells determine the specificity of their EVs. EVs contained RNAs, proteins, and drugs that can play important roles in the development of cancers. EVs have the ability to decide the fate of cells by cell-to-cell communication. EVs have potential applications in anti-cancer treatments in the future.
